# MicroRNA-31: a pivotal oncogenic factor in oral squamous cell carcinoma

**DOI:** 10.1038/s41420-022-00948-z

**Published:** 2022-03-29

**Authors:** Xiaojiao Lin, Weizhou Wu, Yukang Ying, Jun Luo, Xuhui Xu, Linxia Zheng, Weili Wu, Suqing Yang, Shankun Zhao

**Affiliations:** 1 Department of Stomatology, Taizhou Central Hospital (Taizhou University Hospital), 318000 Zhejiang, China; 2 Department of Urology, Maoming People’s Hospital, Maoming, 525000 Guangdong, China; 3 Department of Urology, Taizhou Central Hospital (Taizhou University Hospital), 318000 Taizhou, Zhejiang China

**Keywords:** Pathogenesis, Cancer

## Abstract

Oral squamous cell carcinoma (OSCC) continuously constitutes a major challenge for treatment and prognosis due to approximately half of treated OSCC patients dying from locoregional recurrences and distant metastases. MicroRNA-31 (miR-31), an early mammalian miRNA identified, has been gaining importance in the field of OSCC research in recent years. This comprehensive review was conducted for the first time to summarize the current evidence on the association between miR-31 and OSCC. The vast majority of relevant studies (20/21, 95%) demonstrated that miR-31 was an oncogenic factor in the tumorigenesis and progression of OSCC. miR-31 expression is significantly upregulated in plasma, saliva, and tumor tissue of OSCC. miR-31 played an essential role in OSCC development by constituting a complex network with its targeted genes (e.g. RhoA, FIH, ACOX1, VEGF, SIRT3, LATS2, KANK1, and NUMB) and the signaling cascades (e.g. EGF-AKT signaling axis, ERK-MMP9 cascade, Hippo pathway, Wnt signaling, and MCT1/MCT4 regulatory cascade). This review highlights that miR-31 might function as a potential diagnostic, prognostic, and predictive biomarker for OSCC. Further studies are still warranted to better illuminate the clinicopathological features and the molecular mechanisms of miR-31-mediated OSCC development.

## Facts

- MiR-31 commonly serves as an oncogenic factor in OSCC development.
- MiR-31 level is upregulated in plasma, saliva, and the tumor tissue of OSCC.
- MiR-31 interacts with multiple proteins and pathways that play an essential role in OSCC.

## Open Questions

- What is the exact mechanism of MiR-31 in the pathogenesis of OSCC?
- What are the future clinical implications of MiR-31 examination in OSCC?
- Can we develop an effective drug for OSCC patients by targeting MiR-31?

## Introduction

Oral cancer continues to be one of the leading lethal causes worldwide. According to GLOBOCAN 2020, lip and oral cavity cancer accounted for 377,713 (1.96%) new cases and 177,757 (1.79%) deaths globally [1]. Oral cancer is highly frequent in South Central Asia and Melanesia. Common risk factors for oral cancer include betel nut chewing, consumption of alcohol, tobacco use/cigarette smoking, and HPV infection [1, 2]. Oral squamous cell carcinoma (OSCC) is the most frequent type of malignancies, accounting for more than 90% of all oral cancer cases [3]. The overall 5-year survival rate for OSCC was reported at 50–60% [4], 80 % for the early stage (T1), and 20–40 % at the later stage (T2 or T4) [5]. The therapeutic regimens for OSCC include surgery, radiotherapy, chemotherapy, and immunotherapy. However, approximately half of treated OSCC patients die from locoregional recurrences and distant metastases. Chemotherapy is the main adjuvant therapy for advanced OSCC. However, a chemotherapy commonly does not achieve satisfactory outcomes due to intrinsic and extrinsic resistance, low target selectivity, and serious adverse drugs effects. Motivated by these facts, it is prudent to develop an effective, quick, and non-invasive means to early diagnose and predict the prognosis of OSCC so that minimize the mortality and morbidity of the suffers.

microRNAs (miRNAs) are a class of short, endogenous, non-coding RNA molecules that contain 19-24 nucleotides. miRNAs elicit their biological functions by binding the 3′ untranslated region (UTR) of the target gene mRNA, thus promoting the degradation of the mRNA or leading to translational repression, which eventually implements the post-transcriptional regulation of gene expression [6]. Numerous experimental studies have suggested that miRNAs are involved in the regulation of multiple cellular biological processes, e.g. proliferation, differentiation, and apoptosis. Thus, the dysregulation of miRNAs has been considered to be associated with various pathologies, including the tumorigenesis and progression of OSCC. As reported, a majority of miRNAs have been found to show an aberrant expression in OSCC, i.e., miR-503 [7], miR-210 [8], and miR-146 [9]. Among these miRNAs, microRNA-31 (miR-31) is one of the most investigated miRNAs whose expression undergoes significant changes in OSCC. There has been much research evidence on the roles of miR-31 in tumorigenesis and the development of OSCC. A previous clinical study developed by Liu et al. demonstrated that miR-31 in plasma was significantly increased in patients with OSCC as compared to the age and sex-matched control subjects [10]. In line with this finding, a more recent in *vitro* and in *vivo* study also showed that the level of miR-31 was aberrantly elevated in OSCC cells and tumor tissues and further indicated that miR-31 gene locus was required to elicit oncogenesis in OSCC [11].

Recently, the crucial effect of miR-31 in OSCC has attracted increasing attention from researchers. In this study, we presented a first attempt to summarize all the evidence on the proposed roles of miR-31 in OSCC development via a comprehensive review. Based on the current knowledge, it may be instructive to help the researchers be conscious of the outstanding prognostic and predictive effects of miR-31 on OSCC.

### Overview of miR-31 in cancer

MicroRNA-31 (miR-31), a gene located on chromosome band 9p21.3, was one of the early mammalian miRNAs detected [12]. miR-31 is encoded by a single genomic locus, which can be found in various tissues, cell types, and extracellular exosomes. Consistent with other miRNAs, miR-31 functionally modulates its direct targeted-mRNA via the interaction with the 3′ UTR, constituting the RNA-induced silencing complex thus inducing the silencing of the targeted gene. miR-31 has been found to play role in multiple diseases, e.g. autoimmune diseases, wound healing, and cancer [13]. As reported, miR-31 is among the most commonly varied microRNAs in multiple human malignancies [12], including OSCC [10, 11]. Numerous studies [14] demonstrate that miR-31 participates in cancer pathogenesis and aggressiveness through modulating the target genes. As reported, miR-31 may modulate a series of target genes, including, but not limited to, fibronectin type III domain containing 5 (FNDC5), special AT-rich sequence-binding protein-2 (SATB2), E2F2, large tumor suppressor kinase 2 (LATS2), tensin 1 (TNS1), AT-rich interaction domain 1A (ARID1A), and hypoxia-inducible factor-1 (FIH-1) [15–17]. Commonly, miR-31 is upregulated in the biological processes of cancer [18]. miR-31 expression levels in serum, saliva, urine, and organ tissue can be used as an effective diagnostic and prognostic biomarker of multiple cancers [19, 20]. miR-31 is thought to have high malignant potential because it can dramatically elevate the capability of the migration, growth, and invasiveness of the cancer cells [21]. Currently, miR-31 was proven to be significantly associated with patient survival, response to different treatments, and other clinicopathologic features of multiple cancers, including tumor properties, invasiveness, clinical stage, and metastasis [14, 22]. The above evidence endows miR-31 with an encouraging prospect to be applied as a diagnostic, predictive, and prognostic biomarker in oncological patients. At present, there have been many review articles on the relationship between miR-31 expression and human malignancies, e.g. colorectal cancer, pancreatic cancer, ovarian cancer, prostate cancer [12, 23–26]. However, there is currently no review article that focuses on the clinical significances and molecular mechanisms of miR-31 in OSCC, thus it is worth summarizing all the related evidence on this issue by a comprehensive review.

### Literature search and the characteristic of the included studies

**Fig. 1:**
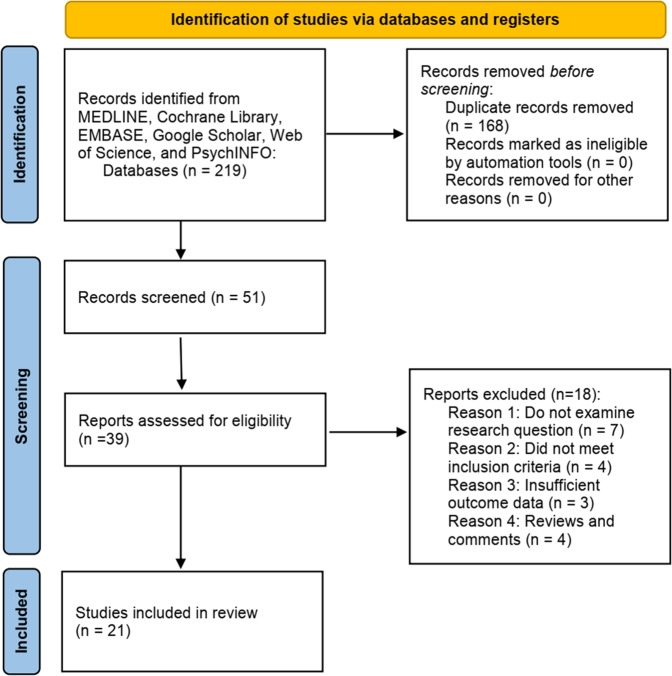
The search flowchart for identifying the eligible studies. Six common-used databases were applied to identify the eligible studies. Finally, 21 studies were included for further reviewing and discussing.

The literature review was undertaken on the six common-used databases, e.g. MEDLINE, EMBASE, Google Scholar, Cochrane Library, Web of Science, and PsychINFO, to discover the related studies reporting the association between miR-31 in OSCC. The searching strategy in the MEDLINE by using the keywords was: ((((miR-31) OR (microRNA-31)) OR (hsa-mir-31)) OR (miR-31-5p)) AND (((((((Oral squamous cell carcinoma) OR (Oral Tongue Squamous Cell Carcinoma)) OR (Hypopharyngeal Squamous Cell Carcinoma)) OR (Oral Cavity Squamous Cell Carcinoma)) OR (Oral Squamous Cell Carcinomas)) OR (Squamous Cell Carcinoma of the Mouth)) OR (Oropharyngeal Squamous Cell Carcinoma)). The reference list was also reviewed so that to identify more relevant studies. Figure 1 displayed the search flowchart for identifying the eligible studies. A conventional data collection table was used to extract the relevant data from the included studies, e.g. the first author’s name and the references, publication years, study/research objects, the role of miR-31, involved mechanism, target gene, associated signaling pathways, and the main findings in each eligible study. Finally, 21 studies [10, 11, 27–45] were included, which were published between 2010 and 2021. The research objects included the tumor tissue, plasma, and saliva of the OSCC patients, OSCC cells, and animal models. Most of the included studies (20/21, 95%) demonstrated that the miR-31 expression was elevated in patients with OSCC. The involved mechanisms mainly included proliferation, migration, invasion, apoptosis, cell cycle, epithelial-to-mesenchymal transition (EMT), cytoskeletal remodeling, reprogramming of lipid metabolism, glycolytic metabolism, oxidative stress, and M2 macrophages dysfunction. The direct targets for miR-31 included ras homolog family member A (RhoA), factors inhibiting HIF (FIH), human telomerase reverse transcriptase (Htert), acyl-CoA oxidase 1 (ACOX1), vascular endothelial growth factor (VEGF), prostaglandin E2 (PGE2), silent information regulator 3 (SIRT3), large tumor suppressor kinase 2 (LATS2), KN motif and ankyrin repeat domain-containing protein 1 (KANK1), wntless (WLS), and NUMB endocytic adaptor protein (NUMB). The associated signaling cascades included GM-CSF signaling, EGF-AKT signaling axis, ERK-MMP9 cascade, Hippo pathway, Wnt signaling pathway, and MCT1/MCT4 regulatory cascade. Table 1 listed the summary of miR-31 in OSCC.

**Table 1:** Summary of miR-31 in oral squamous cell carcinoma (OSCC).

Study/Reference	Research objects	Role of miR-31	Involved mechanism	Target gene	Associated pathways	Main findings
Liu et al. [10]	OSCC patients, plasma and saliva	Up; Oncogenic	Clinical study	Clinical study	Clinical study	Plasma miR-31 was significantly increased in OSCC patients compared to the control subjects (*P* < 0.0001), while 88% (38 ⁄ 43) of the OSCC patients showed a significant degree of decrease in miR-31 level after resection (*P* < 0.0001). Saliva miR-31 was elevated in OSCC patients (*P* = 0.001), while 89% (8/9) exhibited the decrease level after tumor resection.
Liu et al. [27]	Patients plasma and saliva	Up; Oncogenic	Clinical study	Clinical study	Clinical study	Salivary miR-31 was significantly elevated in patients with OSCC at all clinical stages (all *P* < 0.05) compared to the health controls, while the miR-31 expression was significantly reduced after tumor resection; miR-31 was more abundant in saliva than in plasma.
Chang et al. [28]	Tissues and oral cells	Up; Oncogenic	Proliferation, migration	RhoA	NA	miR-31 and miR-31* was up-regulated in OSCC tissues, the activity of miR-31*’s activity counteracted the functions of miR-31 during OSCC tumorigenesis.
Siow et al. [29]	Patients tissue	Up; Oncogenic	Cell cycle; cytoskeletal remodeling; EMT	Unknown	Regulation of G1/S transition; GM-CSF signaling ↑	miR-31 was significantly increased in OSCC tissue (*P* < 0.05) and significantly associated with TNM staging and site (*P* < 0.05).
Lu et al. [30]	Tissues and oral cells	Up; Oncogenic	NA	Unknown	EGF-AKT signaling axis ↑	EGF up-regulated miR-31 expression via the AKT pathway, EGFR-AKT-C/EBPβ regulatory axis may underlie miR-31 up-regulation in OSCC.
Hung et al. [31]	Tissues and Oral keratinocytes	Up; Oncogenic	Proliferation, migration, and EMT	FIH, hTERT, and VEGF	NA	Increased miR-31 level was observed in oral potentially malignant disorder tissues; miR-31 expression contributed to the acquirement of the EMT by M31OK1 cells; Oral tumorigenesis correlated to the upregulation of miR-31 targeted FIH, hTERT, and VEGF.
Kolokythas et al. [32]	Patients tissues	Up; Oncogenic	Clinical study	Clinical study	Clinical study	miR-31-5p was enriched in the tumor epithelium in OSCC compared to benign pathology.
Severino et al. [33]	Plasma	Up in non-metastatic samples	Clinical study	Clinical study	Clinical study	miR-31 was over-expressed in non-metastatic samples of OSCC compared with the metastatic samples.
Kao et al. [34]	Mouse and cell	Up; Oncogenic	NA	Unknown	EGFR/AKT/S6	Salivary and plasma miR-31 increased along with the development of tongue carcinogenesis; The increase of salivary miR-31 was higher than in plasma; miR-31 might activate EGFR/AKT/S6 or enhance the oncogenic process for the subsequent tumor induction.
Cinpolat et al. [35]	Patients tissues	Up; Oncogenic	Clinical study	Clinical study	Clinical study	miR-31 was up-regulated in the tissues of salivary gland tumor group compared to benign group (*P* = 0.02).
Yan et al. [36]	Patients tissues	Up; Oncogenic	NA	Unknown	NA	miR-31 was identified to play essential roles in carcinogenesis of OSCC.
Lai et al. [37]	Patients tissues, OSCC cell lines	Up; Oncogenic	Reprogramming of lipid metabolism; migration, invasion	ACOX1	ERK-MMP9 cascade ↑	miR-31-5p was a highly expressed miRNA in OSCC; miR-31-5p-ACOX1- PGE2 axis positively affect the extent of cell motility in correlation with metastatic status of OSCC.
Yap et al. [38]	Patients tissues	Up; Oncogenic	Clinical study	Clinical study	Clinical study	Upregulation of miR-31 was found in OSCC in both formalin-fixed paraffin embedded and fresh frozen samples.
Kao et al. [39]	OSCC tissues and cells	Up; Oncogenic	Glycolytic metabolism, oxidative stress, migration, invasion	SIRT3	NA	miR-31 involved in ROS regulation and OSCC cells invasion by affecting SIRT3 expression; SIRT3 expression reduced the tumorigenicity and disrupted mitochondrial structure of OSCC cells; miR-31 inhibited the respiratory activity and elevated lactate production in OSCC cells.
Jakob et al. [40]	Patients tissues	Up; Oncogenic	Clinical study	Clinical study	Clinical study	miR-31 was significantly upregulated in OSCC when compared to the controls (*P* < 0.001).
Jung et al. [41]	OSCC cell lines and drosophila melanogaster	Down; Tumor-suppressive	Cell cycle and proliferation	WLS	Cyclin D1, c-MYC, and Wnt signaling pathway ↓	miR-31-induced suppression of tissue growth; Overexpression of miR-31 in OSCC cells induced downregulation of WLS, a putative target for miR-31, together contributing to suppress tumor growth, suggesting miR-31 might be a tumor suppressor.
Peng et al. [42]	OSCC tissues and cells	Up; Oncogenic	Proliferation, migration, invasion, and EMT	LATS2	Hippo signaling pathway ↓	miR-31 level was markedly increased in OSCC tissues; circ_0000140 negatively associated with miR-31 expression (*r*^2^ = 0.43, *P* < 0.001) and positively associated with LATS2 expression (*r*^2^ = 0.60, *P* < 0.001).
Wang et al. [43]	OSCC tissues and cells	Up; Oncogenic	Apoptosis, cisplatin sensitivity	KANK1	Long non-coding RNA CASC2- miR-31-5p/KANK1 axis ↑	miR-31-5p was upregulated in cisplatin-resistant OSCC tissues and cells, KANK1 acted as a target for miR-31-5p; CASC2 modulated KANK1 expression via sponging miR-31-5p.
Kumari et al. [44]	Patients saliva	Up; Oncogenic	Clinical study	Clinical study	Clinical study	The salivary miR-31 level was significantly higher in the preoperative patients than that of postoperative (*P* < 0.001). miR-31 might be a potential non-invasive marker to monitor surgery outcomes during postoperative follow-up in patients with OSCC.
Chou et al. [11]	OSCC cell and Mouse	Up; Oncogenic	Proliferation, migration, invasion	NUMB	MCT1/MCT4 regulatory cascade ↑	miR-31 gene locus was required to elicit oncogenesis in OSCC cells, while NUMB was the target of miR-31; Reduced NUMB expression upregulated MCT1/MCT4 level; MCT1 or MCT4 expression in tumors was associated with worse survival;
Yuan et al. [45]	Patients tissues, mouse, and cell	Up; Oncogenic	Proliferation, tumorigenesis, M2 macrophages dysfunction	LATS2	Hippo signaling pathway ↓	M2 macrophage-derived exosomal miR-31-5p might inhibited the tumor suppressor LATS2, thus facilitating the progression of OSCC via suppressing the Hippo signaling pathway.

*OSCC* oral squamous cell carcinoma, *RhoA* ras homolog family member A, *EMT* epithelial-to-mesenchymal transition, *FIH* factors inhibiting HIF, *hTERT* human telomerase reverse transcriptase, *VEGF* vascular endothelial growth factor, *PGE2* prostaglandin E2, *ACOX1* acyl-CoA oxidase 1, *ROS* reactive oxygen species, *SIRT3* silent information regulator 3, *LATS2* large tumor suppressor kinase 2, *KANK1* KN motif and ankyrin repeat domain-containing protein 1, *CASC2* long non-coding RNA cancer susceptibility candidate 2, *WLS* wntless, *NUMB* NUMB endocytic adaptor protein.

### Clinical significances of miR-31 IN OSCC

There are fourteen included studies that provided detailed clinical information of miR-31 expression level in OSCC. All the eligible clinical studies reported that miR-31 was upregulated in OSCC patients.

### miR-31 expression was higher in malignant tissues than benign tissues

Significant histopathological alterations of miR-31 expression observed in the tissue sections between OSCC and benign tissue can intuitively reflect the critical roles of miR-31 in OSCC development and progression. All the included studies that provided the information on the expression of miR-31 in pathological sections consistently suggested that the level of miR-31 was up-regulated in OSCC tissues. But the association between miR-31 and the clinical characteristics of OSCC was a bit different among different studies. Siow et al. [29] found that miR-31 level was significantly associated with TNM staging and site of OSCC (all *P* < 0.05). Kolokythas et al. [32] reported that miR-31-5p was enriched in the tumor epithelium in OSCC compared to benign pathology. miR-31 was also found to be correlated with the metastatic status of OSCC [37]. A previous study indicated that miR-31 upregulation was observed in OSCC in both formalin-fixed paraffin-embedded and fresh frozen samples [38]. Moreover, miR-31 was also identified to be associated with the chemotherapy resistance of OSCC treatments. For example, Wang et al. [43] found that miR-31-5p was upregulated in cisplatin-resistant OSCC tissues. The above evidence demonstrated that high expression of miR-31 was closely associated with the clinicopathologic features of OSCC, e.g. TNM staging, tumor site, and chemotherapy resistance.

### The plasma and saliva miR-31 was significantly increased in patients with OSCC, which was associated with the clinical features of OSCC

As reported, increased expression of miR-31 was not only observed in the OSCC tissues, but also the plasma and saliva of an OSCC patient. Numerous studies have demonstrated that miRNAs have the potential to be detected as effective biomarkers in body fluids. Liu et al. [10] reported that plasma miR-31 was dramatically elevated in OSCC patients compared to the control subjects (*P* < 0.0001), while 88% (38/43) of the OSCC patients showed a significant degree of decrease in miR-31 level after resection (*P* < 0.0001). Consistently, the authors also found that saliva miR-31 was elevated in OSCC patients compared to the non-cancer subjects (*P* = 0.001), while 89% (8/9) exhibited the decreased level after tumor resection. In the subsequent study [27], the authors further indicated that the salivary miR-31 was significantly increased in patients with OSCC at all clinical stages (all *P* < 0.05), while the miR-31 expression was remarkably reduced after tumor resection. Besides, Liu et al. demonstrated that miR-31 was more abundant in saliva than in plasma. Kumari et al. [44]revealed that the salivary miR-31 level was significantly higher in the preoperative patients than that of postoperative (*P* < 0.001), suggesting miR-31 might be a potential non-invasive marker to monitor surgery outcomes during postoperative follow-up in patients with OSCC. Higher levels of miR-31 level have frequently been found in OSCC patients than cancer-free individuals, while miR-31 expression in metastatic- and non-metastatic OSCC is rarely reported. A study [33] conducted in Brazil showed that plasma miR-31 was overexpressed in non-metastatic samples of OSCC than that of metastatic samples. This study pinpointed the fact that plasma miR-31 was higher in OSCC patients than the healthy controls, while its expression was higher in patients with non-metastatic OSCC when compared to those with metastasis.

### Molecular mechanisms of miR-31 in OSCC

Due to the aforementioned clinical studies having suggested a causal relationship between miR-31 level and OSCC, a better understanding of the biological functionings of miR-31 and its underlying mechanisms in OSCC tumorigenesis and development is profound for the investigators. miR-31 was up-regulated in the majority of OSCC and thus it was thought to play an oncogenic role in OSCC.

### Cell cycle regulation underlies the effect of miR-31 in OSCC tumorigenesis

Cell cycle control is one of the crucial cancer-related pathways in the development of multiple malignancies, including OSCC. Altered expression of miRNAs can result in the inhibition or promotion of cell cycle arrest and cell death. Experimental studies have demonstrated that miR-31 was involved in the progression and metastasis of various types of cancers by regulating the cell cycle [46, 47]. Siow et al. [29] reported that elevation of miR-31 expression was correlated with the clinical features in OSCC. The carcinogenic mechanisms of miR-31 in OSCC were supposed to the regulation of the cell cycle via the G1/S transition. Jung et al. [41] also indicated that the cell cycle might participate in the action of miR-31 in OSCC development. However, in contrast to Siow et al.’s findings, Jung et al. demonstrated that miR-31 might serve as a tumor suppressor in OSCC due to the level of miR-31 could induce the suppression of OSCC tumor growth. The authors speculated that the underlying mechanisms of miR-31 inhibiting OSCC might be associated with the down-regulation of the driving factors of the cell cycle, e.g. Cyclin D1 and c-MYC. In addition, this study also suggested that wntless (WLS), a putative target for miR-31, together contribute to OSCC suppression by regulating the Wnt signaling pathway [41].

### Roles of EMT in miR-31-regulated OSCC

EMT, a widely accepted mechanism for cancer development, occurs frequently during the pathological process associated with tumorigenesis and cancer progression towards metastasis [14]. Migratory and invasive behaviors of the cancer cells may encounter an enhancement after a shift towards the mesenchymal state of the malignant cells, which are characterized by the abnormal expression of EMT-related molecules and the shape cells altering into spindle [48]. Numerous studies suggest that miR-31 exhibits the oncogenic effect on multiple cancers through the EMT process [49]. In the present review, three studies [29, 31, 42] demonstrated that EMT might be one of the key pathomechanisms underlying the miR-31-mediated OSCC. All these studies indicated the expression of miR-31 was increased in OSCC patients compared to the non-tumoral individuals. Hung et al. [31] reported that up-regulation of miR-31 contributed to the acquirement of the EMT by M31OK1 cells. They further found that oral tumorigenesis might correlate to the elevation of miR-31 targeted genes, e.g. factors inhibiting HIF (FIH), human telomerase reverse transcriptase (hTERT), and vascular endothelial growth factor (VEGF). Peng et al. [42] revealed that miR-31 expression was markedly increased in OSCC tissues, while circ_0000140 negatively associated with miR-31 level (*r*^2^ = 0.43, *P* < 0.001) and positively associated with large tumor suppressor kinase 2 (LATS2) expression (*r*^2^ = 0.60, *P* < 0.001). The authors also found that the Hippo signaling pathway played an essential role in this action. The above evidence indicated that EMT might greatly contribute to tumorigenesis and the progression of miR-31-associated OSCC.

### miR-31 functions as an oncogenic factor by increasing the proliferative, migratory, and invasive capacities of the OSCC cells

Proliferation, migration, and invasion strongly correlate with a malignant phenotype. According to a large body of experimental studies, multiple miRNAs play crucial roles in cancer development by strengthening the proliferative, migratory, and invasive capacities of the cancer cells [50, 51]. Chang et al. [28] reported that miR-31 promoted the proliferation and migration of oral cancer cells by targeted RhoA. In agreement with Chang et al.’s findings, Hung et al. [31] also demonstrated that up-regulated miR-31 could induce OSCC tumorigenesis via the enhancement of proliferation and migration. There were also several studies [37, 39] indicating the oncogenic effects developed by miR-31 were largely dependent on the promotion of migration and invasion of the oral cancer cells. Lai et al. [37] reported that miR-31-5p positively affected the extent of cell motility in correlation with the metastatic status of OSCC by targeting ACOX1 and upregulating ERK-MMP9 cascade. Kao et al. [39] found that miR-31 was involved in OSCC cells migration and invasion by affecting SIRT3 expression. Besides, there have been many studies [11, 42] that suggested that the roles of miR-31 in OSCC might be associated with the elevation of proliferation, migration, and invasion of the OSCC cells which mainly were mediated the targeted genes. Peng et al. [42] revealed that circ_0000140 was negatively associated with miR-31 expression (*r*^2^ = 0.43, *P* < 0.001) and positively associated with the miR-31-targeted LATS2 expression (*r*^2^ = 0.60, *P* < 0.001). A more recent study [11] demonstrated that miR-31 gene locus was required to elicit oncogenesis in OSCC cells. NUMB, a direct target of miR-31, was negatively associated with the level of MCT1/MCT4 which upregulation in tumors was found to correlate with worse survival.

### Other potential mechanisms underlay miR-31-mediated OSCC

As shown in Table 1, in addition to the above-supposed mechanisms, other pathomechanisms included cytoskeletal remodeling, reprogramming of lipid metabolism, glycolytic metabolism dysfunction, oxidative stress injury, anti-apoptosis, M2 macrophages dysfunction, and the dysfunction of affected signaling pathways. Both glycolytic metabolism and oxidative stress are considered to involve in the pathogenesis and progression of multiple malignancies [52]. Kao et al. [39] suggested that miR-31 participated in OSCC development by disrupting the mitochondrial structure and increasing lactate production in OSCC cells. Cisplatin is one of the common-used first-line chemotherapy regimens for treating OSCC but usually fails because of chemoresistance [53]. miR-31-5p upregulation was found in cisplatin-resistant OSCC, which was considered to be associated with the anti-apoptosis characteristic of miR-31 [43]. Tumor-associated macrophages are the crucial elements of the tumor microenvironment. M2 macrophage-derived exosomes were found to promote cancer progression by delivering miRNA [54]. Yuan et al. [45] demonstrated that macrophage-derived exosomal miR-31-5p might inhibit the tumor suppressor LATS2, thus facilitating the progression of OSCC via suppressing the Hippo signaling pathway. Various lines of evidence suggest that the basic leucine zipper transcription factor CCAAT/enhancer binding protein β (C/EBPβ) is an oncogenic factor [55]. The epidermal growth factor receptor (EGFR) signaling pathway is considered to play an important role in driving the oncogenesis of OSCC by triggering various proteins and intracellular signaling networks (i.e., AKT) [56, 57]. Lu et al. [30] reported that EGF might up-regulate the level of miR-31 expression through the AKT signaling cascade in a malignant phenotype of OSCC. The author further found that the C/EBPβ cascade could increase the expression of miR-31 and there was a significant correlation between C/EBPβ and miR-31 expression during OSCC pathogenesis.

**Fig. 2:**
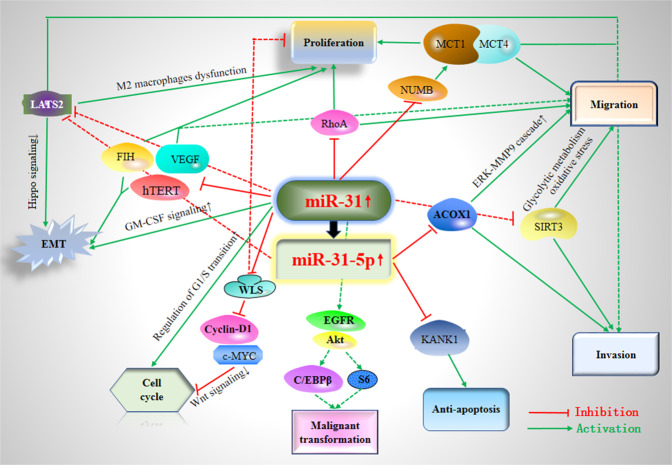
Main mechanisms of miR-31 in oral squamous cell carcinoma (OSCC). miR-31 exerts its central roles in OSCC by constituting a complex network with the direct target genes (e.g. RhoA, FIH, ACOX1, VEGF, SIRT3, LATS2, KANK1, and NUMB) and the signaling cascades (e.g. ERK-MMP9 cascade, Hippo pathway, Wnt signaling, and MCT1/MCT4 regulatory cascade). RhoA= ras homolog family member A; EMT = epithelial-to-mesenchymal transition; FIH = factors inhibiting HIF; hTERT = human telomerase reverse transcriptase; VEGF = vascular endothelial growth factor; PGE2 = prostaglandin E2; ACOX1 = acyl-CoA oxidase 1; ROS = reactive oxygen species; SIRT3 = silent information regulator 3; LATS2 = large tumor suppressor kinase 2; KANK1 = KN motif and ankyrin repeat domain-containing protein 1; CASC2 = long non-coding RNA cancer susceptibility candidate 2; WLS = wntless; NUMB = NUMB endocytic adaptor protein.

**Fig. 3:**
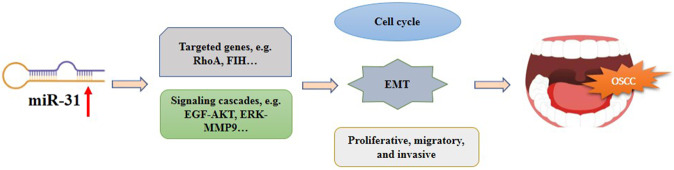
Diagram of the main findings of this review. Upregulation of miR-31 involves in the development OSCC by interacting with the targeted genes and signaling cascades that affect the cell cycle, EMT, and cell growth of the cancer cells.

Figure 2 illustrated the detailed potential molecular mechanisms of miR-31 in OSCC. Figure 3 summarized the main findings of this review via a diagram.

## Conclusion and perspectives

To the best of our knowledge, the present comprehensive review is the first study that summarizes all the current evidence on the association between miR-31 and OSCC. The vast majority of relevant studies demonstrate that miR-31 is an oncogenic factor in the tumorigenesis and progression of OSCC. miR-31 expression is significantly upregulated in plasma, saliva, and tumor tissue of OSCC, which enhances the malignant phenotypes of OSCC. This review highlights that miR-31 may function as a potential therapeutic target owing to its essential role in OSCC development. miR-31 interacts with multiple signaling cascades by binding to its targeted proteins, constituting a complex network that promotes OSCC. In the future, a better understanding of the association of miR-31 with clinicopathological features and the molecular mechanisms could provide insights into the crucial role of miR-31 in driving OSCC malignant transformation, which may favor the development of miR-13-based diagnostic, prognostic, and predictive biomarker for OSCC.

## Data Availability

The data used to support the findings of this study are available from the corresponding author upon request.
